# Atypical Visceral Involvement of Pyoderma Gangrenosum

**DOI:** 10.31486/toj.25.0094

**Published:** 2026

**Authors:** Nicolás Martínez Ruiz, Javier Oliva Ibarz, Saray Rodríguez López, Justyna Szafranska, Sheila Alfonso-Cerdán

**Affiliations:** ^1^ Department of Radiology, Hospital de la Santa Creu i Sant Pau, Barcelona, Spain; ^2^ Department of Pathology, Hospital de la Santa Creu i Sant Pau, Barcelona, Spain

**Keywords:** *Abdominal abscess*, *abdominal pain*, *colitis–ulcerative*, *infliximab*, *paraneoplastic syndromes*, *pyoderma gangrenosum*, *skin diseases*

## Abstract

**Background:**

Pyoderma gangrenosum is a noninfectious reactive inflammatory dermatosis included in the spectrum of neutrophilic dermatoses. Extracutaneous manifestations of pyoderma gangrenosum are rare, but when visceral lesions occur, they are typically detected through radiologic tests, can be cavitated or noncavitated, and progress rapidly. Because of the difficult etiologic diagnosis of these lesions, the use of nonproductive and potentially harmful invasive procedures and treatments is common.

**Case Report:**

A 58-year-old female with a history of ulcerative colitis presented to the emergency department with a history of pain in the right sternoclavicular joint radiating to the shoulder. Cervicothoracic computed tomography (CT) suggested right sternoclavicular arthritis. During hospitalization, the patient experienced a flare of ulcerative colitis and developed cutaneous lesions. The patient worsened clinically with increased abdominal pain in the left hemiabdomen and elevated acute phase reactants. Thoracoabdominal CT revealed a hypodense lesion on the anterior aspect of the spleen that was deemed a splenic abscess, and ultrasound-guided percutaneous drainage was placed. Cultures were negative. Subsequently, 2 cutaneous lesions appeared on the patient's right arm, suggestive of abscesses related to attempts to place a midline catheter. All cultures were negative. Given the negative microbiologic cultures and the appearance of cutaneous lesions, pyoderma gangrenosum with visceral involvement was diagnosed and the patient was successfully treated with infliximab.

**Conclusion:**

While visceral involvement of pyoderma gangrenosum is uncommon, correct diagnosis is crucial to avoid unnecessary treatments and interventions.

## INTRODUCTION

Pyoderma gangrenosum is a noninfectious reactive inflammatory dermatosis included in the spectrum of neutrophilic dermatoses.^1,2^ The usual clinical manifestation is cutaneous lesions that develop as painful, edematous nodules or vesicles that progress to ulcers with violaceous and edematous margins.^1,2^ The incidence is estimated to be 3 to 10 cases per million population per year.^3^

Pyoderma gangrenosum is associated with other systemic diseases and, in adults, often coexists with inflammatory, hematologic, or malignant diseases as a paraneoplastic syndrome.^1^ Between 50% and 70% of adult patients have an underlying disease at the time of diagnosis.^1^ While extracutaneous manifestation is rare,^1^ when visceral lesions occur, they can be cavitated or noncavitated; progress rapidly; and mimic neoplasms, abscesses, deep fungal infections, granulomatosis with polyangiitis, or tuberculosis.^2,4-6^

We present the case of a patient who was initially suspected of having a splenic abscess but was ultimately diagnosed with pyoderma gangrenosum with visceral involvement.

## CASE REPORT

A 58-year-old female with a history of smoking, ulcerative colitis treated with mesalazine, and breast cancer in remission for 6 years presented to the emergency department (ED) with a 10-day history of pain in the right sternoclavicular joint radiating to the shoulder. The patient had a fever of 38 °C. Blood tests revealed an increase in acute phase reactants: leukocytes of 12,600 × 10^9^/L (reference range, 3,800-11,000 × 10^9^/L) and C-reactive protein of 270 mg/L (reference range, 0-5 mg/L).

**Figure 1.**
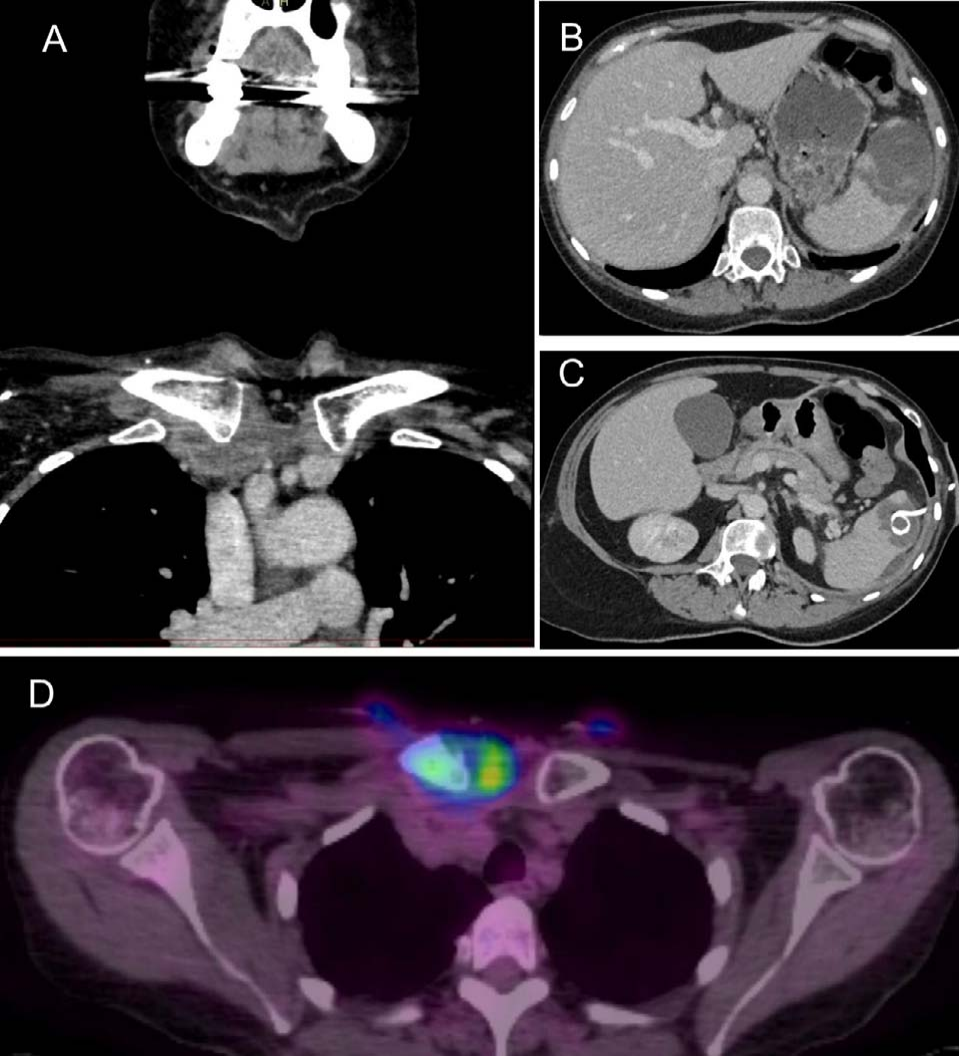
(A) Coronal reformation of a contrast-enhanced computed tomography (CT) scan shows thickening and hyperenhancement of the synovium of the right sternoclavicular joint with associated joint effusion. (B) Axial contrast-enhanced CT image shows a hypodense lesion located along the anterior aspect of the spleen before percutaneous drainage. (C) Axial contrast-enhanced CT image obtained 3 days after percutaneous drainage shows a marked reduction in lesion size. (D) Axial positron emission tomography image shows hypermetabolism in the right sternoclavicular joint and adjacent soft tissues.

Cervicothoracic computed tomography (CT) and positron emission tomography (PET) CT suggested right sternoclavicular arthritis with contrast enhancement of the synovium, joint effusion, and hypermetabolic uptake (Figures 1A and 1D). This finding led to the patient's admission and initiation of broad-spectrum antibiotics (piperacillin/tazobactam 4 g every 6 hours intravenously [IV] for 10 days) and corticosteroids (prednisone 30 mg every 24 hours for 5 days), as well as joint debridement.

On day 4 of hospitalization, the patient experienced a flare of ulcerative colitis, characterized by 8 to 10 bowel movements per day, and pathological diarrhea with blood and mucus. Treatment with methylprednisolone was started (20 mg every 8 hours IV for 7 days). Colonoscopy with biopsy performed on day 6 of hospitalization demonstrated active ulcerative proctitis.

One week after admission, the patient developed cutaneous lesions—red blisters on the trunk and arms—suspected to be secondary to herpes zoster, and treatment with oral acyclovir (800 mg 5 times daily for 7 days) was initiated. The next day, the patient worsened clinically with increased abdominal pain in the left hemiabdomen and elevated acute phase reactants. Thoracoabdominal CT scan revealed a hypodense lesion measuring 62 × 60 mm on the anterior aspect of the spleen (Figure 1B). Initially, the lesion was considered to be a splenic abscess. On day 9 of admission, the patient further deteriorated clinically, with elevated inflammatory markers (leukocytes of 18,700 × 10^9^/L and C-reactive protein of 310 mg/L), fever of 38.6 °C, and tachycardia of 122 bpm. On day 10, an ultrasound-guided percutaneous pigtail catheter was placed and drained hematic fluid. Follow-up CT imaging 3 days after the catheter was placed showed a size reduction of the hypodense splenic lesion (Figure 1C). Cultures from the drained fluid were negative. No additional tests were performed on the sample.

On day 12 of hospitalization, 2 cutaneous lesions appeared on the patient's right arm, suggestive of abscesses related to attempts to place a midline catheter 5 days earlier. On day 14, both lesions were debrided under local anesthesia. The samples were not sent to pathology but were analyzed microbiologically, with no microbial growth detected. Treatment with broad-spectrum antibiotics (piperacillin/tazobactam 4 g every 6 hours IV for 1 week) was restarted, and methylprednisolone was discontinued because of the risk of worsening the septic condition. Cultures taken from the midline puncture site were negative.

Given the negative microbiologic cultures (both splenic and cutaneous) and the appearance of the cutaneous lesions, the patient was referred to Dermatology. Their diagnostic hypothesis was pyoderma gangrenosum with visceral involvement, and treatment with the monoclonal antibody infliximab (5 mg/kg IV) was initiated as induction therapy, with a first dose on day 16 and a second dose on day 30 of hospitalization. Two to 3 days after treatment initiation, the patient's symptoms markedly improved: diarrhea decreased and fever resolved. Cutaneous lesions resolved after 5 days, and acute phase reactant values in follow-up blood testing were normal (leukocytes of 8,000 × 10^9^/L and C-reactive protein of 4.8 mg/L). The patient was discharged 4 weeks after admission. A third dose of infliximab was administered on an outpatient basis 2 weeks after discharge, and maintenance therapy was administered every 8 weeks. Infliximab maintenance treatment was discontinued after 8 months because of clinical remission. Follow-up was limited, as the patient moved to another city a few months after the discharge.

Two years after the described episode, after 16 months off therapy, the patient presented to a primary care center with complaints of general malaise, fever of 39 °C, and left hypochondrial pain, accompanied by 2 to 3 daily diarrheal stools without pathological products of blood and mucus. Initially, the condition was considered acute gastroenteritis, and symptomatic treatment (fluid therapy and oral acetaminophen 1 g every 8 hours) was indicated. Because her symptoms did not improve and she developed blistering skin lesions in the right gluteal region, the patient sought care at a hospital ED 2 days after visiting the primary care center.

**Figure 2.**
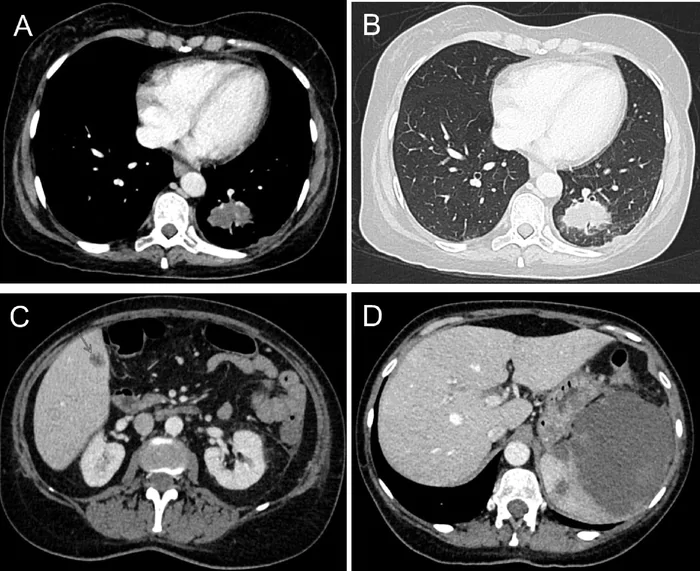
Axial contrast-enhanced computed tomography (CT) scans show a consolidative lesion in the left lower lobe on (A) mediastinal and (B) lung window settings. (C) A slightly hypodense lesion is identified in hepatic segment V (arrow). (D) A hypodense splenic lesion is observed in the same location as on the CT scan performed 2 years earlier (Figure 1B), although with an increase in size.

**Figure 3.**
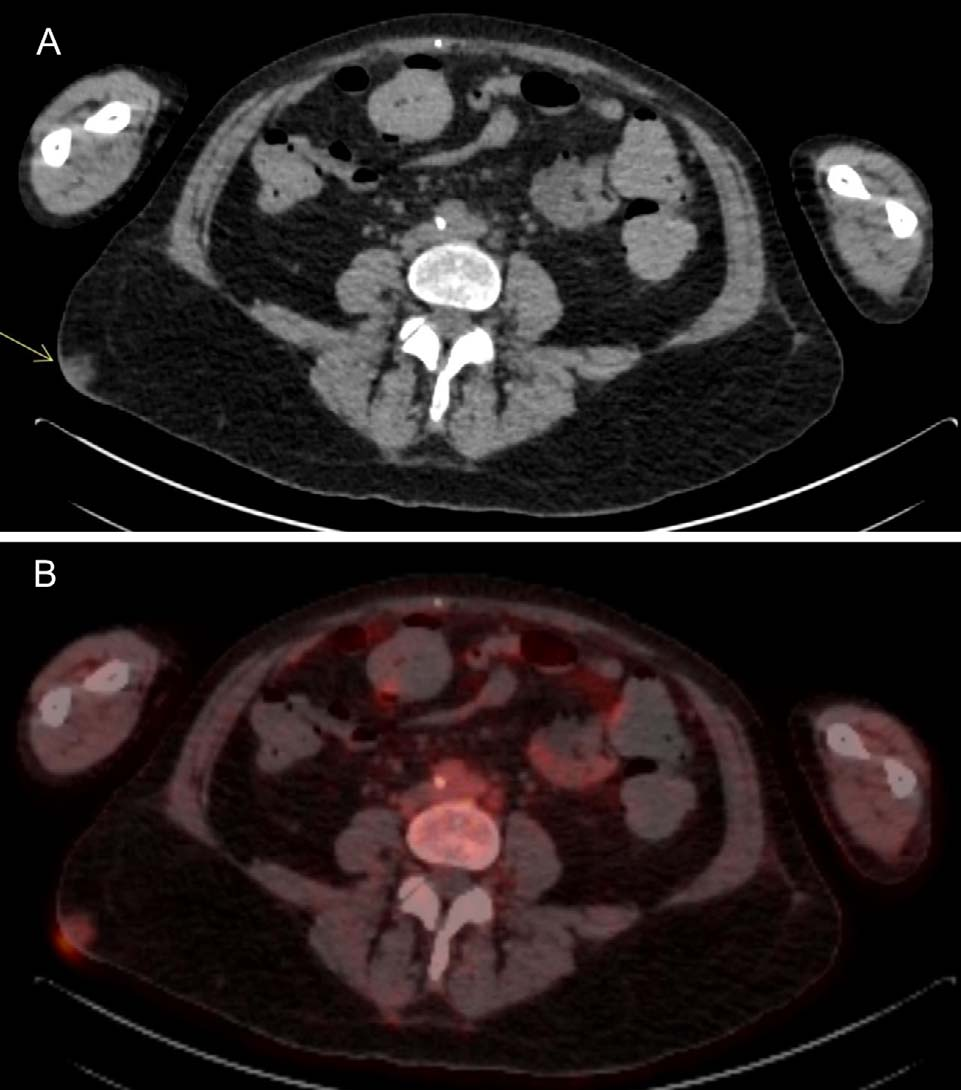
(A) Axial noncontrast-enhanced computed tomography scan shows a cutaneous-subcutaneous lesion in the right lateral lumbo-gluteal region. (B) Corresponding positron emission tomography image shows increased metabolic activity of the same lesion.

Thoracoabdominal CT scan revealed a nodular consolidation in the lower lobe of the left lung, a focal nodular hypodense lesion in hepatic segment V, and an increase in the size of the previously identified splenic lesion (Figure 2). PET-CT showed a hypermetabolic cutaneous-subcutaneous lesion in the right lateral lumbo-gluteal region (Figure 3) compatible with pyoderma gangrenosum. This lesion was identified on targeted physical examination and subsequently biopsied.

**Figure 4.**
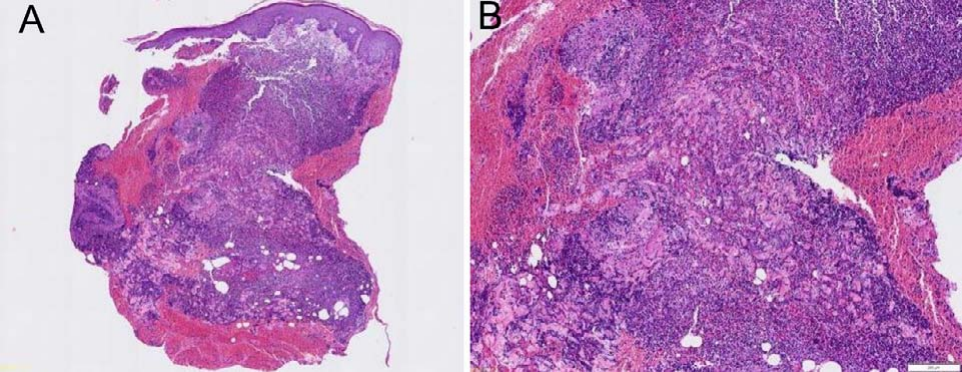
Histopathologic examination of the biopsy specimen from the cutaneous lesion in the right lumbo-gluteal region at (A) lower magnification and (B) higher magnification showed focal epidermal ulceration, a marked mixed dermal inflammatory infiltrate with abscess formation, focal necrotizing granulomas, and occasional multinucleated giant cells. No microorganisms or histologic evidence of malignancy were observed. Special stains for fungi, acid-fast bacilli, and bacteria were negative. Overall, the histopathologic findings were consistent with the clinical suspicion of pyoderma gangrenosum.

The biopsy revealed focal epidermal ulceration, a marked mixed dermal inflammatory infiltrate with abscess formation, the presence of focal necrotizing granulomas, and occasional multinucleated giant cells (Figure 4). No microorganisms or histologic signs of malignancy were observed. Special stains for fungi (periodic acid-Schiff and Grocott methenamine silver), acid-fast bacilli (Ziehl-Neelsen), and bacteria (Gram) were negative. The histologic findings were consistent with the clinical suspicion of pyoderma gangrenosum.

Considering the patient's history, the results of the radiologic and histologic tests, the appearance of the lesion, the coincidence with an ulcerative colitis flare, and the negative infection tests, the clinical case was interpreted as a new flare of pyoderma gangrenosum with multiple visceral involvement (hepatic, splenic, and pulmonary), and the patient was admitted to the hospital. Surgical debridement was avoided, and induction treatment with infliximab was initiated (5 mg/kg IV), with a first dose administered on day 6 and a second dose on day 20 of hospitalization. The treatment led to a rapid clinical improvement with easing of the diarrhea and arthralgias and fever resolution within 4 days. The patient was discharged 3 weeks after admission on infliximab maintenance treatment and with instructions for Dermatology and Rheumatology follow-up. A third dose of induction infliximab was administered on an outpatient basis 3 weeks after discharge.

## DISCUSSION

Pyoderma gangrenosum typically presents as a rapidly enlarging necrotic ulcer in the dermis and can trigger systemic symptoms.^2^ Our case is an example of this possibility, as the patient's cutaneous manifestation was associated with systemic disease characterized by fever; generalized weakness; joint involvement; and visceral involvement affecting the liver, spleen, and lungs.

Few cases of extracutaneous complications of pyoderma gangrenosum have been reported.^1-10^ Various organs have been affected, including the lungs, lymph nodes, bones, liver, spleen, and kidneys.^2^ Pulmonary involvement is the most common visceral complication,^2^ typically manifesting as massive neutrophilic infiltrates or cavitated lesions associated with pleural effusion and presenting with symptoms such as dyspnea, cough, and fever. Hepatosplenic involvement is less frequent and is often initially diagnosed as an infectious process.^5^ Consequently, empiric antibiotic therapy is generally ineffective. In the setting of persistently negative cultures, the diagnosis is reconsidered in favor of a possible neutrophilic dermatosis such as pyoderma gangrenosum, as in our case, given the well-recognized propensity of pyoderma gangrenosum to mimic infection.

Correct diagnosis is crucial to avoid unnecessary treatments and interventions and to ensure that appropriate therapy is administered. Because of the rarity of pyoderma gangrenosum, no standardized treatment protocol has been established, and therapeutic decisions are largely guided by case series and expert opinion.^3,10^ Treatment of pyoderma gangrenosum is immune modulation generally with prednisone, azathioprine, or cyclosporine.^6,10^ Clinical improvement is typically observed within days to weeks.

While prednisone is widely regarded as a first-line treatment for pyoderma gangrenosum with visceral manifestations, variable responses have been reported, with some patients demonstrating limited or no response to corticosteroids.^11^ Infliximab, a monoclonal antibody that inhibits tumor necrosis factor alpha, has shown therapeutic benefit in this setting.^9^ In a randomized, double-blind, placebo-controlled trial of patients with cutaneous pyoderma gangrenosum, patients treated with infliximab demonstrated a rapid and significant clinical response vs patients treated with placebo.^12^

## CONCLUSION

The association of pyoderma gangrenosum with underlying inflammatory conditions is well known. Although pyoderma gangrenosum is a rare condition, awareness of potential extracutaneous involvement is key for accurate diagnosis and appropriate treatment. Clinicians should consider pyoderma gangrenosum in patients who present with visceral lesions that mimic abscesses, particularly when microbiologic cultures are negative and the patient does not respond to appropriate antibiotic therapy.

## Notes

*This article meets the Accreditation Council for Graduate Medical Education and the American Board of Medical Specialties Maintenance of Certification competencies for Patient Care and Medical Knowledge.*

## References

[1] Kędzierska MA, Szmurło A, Szymańska E, Walecka I. Pyoderma gangrenosum with visceral involvement: a severe, recurrent disease affecting pulmonary, splenic, mesorectal, and subcutaneous tissues. Pol Arch Intern Med. 2020;130(7-8):688-690. doi: 10.20452/pamw.15368 32426955

[2] Borda LJ, Wong LL, Marzano AV, Ortega-Loayza AG. Extracutaneous involvement of pyoderma gangrenosum. Arch Dermatol Res. 2019;311(6):425-434. doi: 10.1007/s00403-019-01912-1 30923901

[3] Monari P, Moro R, Motolese A, Epidemiology of pyoderma gangrenosum: results from an Italian prospective multicentre study. Int Wound J. 2018;15(6):875-879. doi: 10.1111/iwj.12939 29877043 PMC7949684

[4] Bansal A, Singla A, Singh A, Kaur S. Pyoderma gangrenosum with splenic abscess – a rare association. Indian Dermatol Online J. 2022;13(2):244-247. doi: 10.4103/idoj.idoj_274_21 35287426 PMC8917506

[5] Sitjas D, Llistosella E, Peñarroja G, Castro A, Codina-Barreras A. Pyoderma gangrenosum with hepatosplenic and joint involvement. Article in Spanish. Actas Dermosifiliogr. 2004;95(10):641-643. doi: 10.1016/S0001-7310(04)76904-5

[6] Cosgarea R, Senilă SC, Badea R, Ungureanu L. Pyoderma gangrenosum with spleen involvement. Review of the literature and case report. J Dermatol Case Rep. 2016;10(2):26-31. doi: 10.3315/jdcr.2016.1230 27900062 PMC5124368

[7] Batalla A, Pérez-Pedrosa A, García-Doval I, González-Barcala FJ, Rosón E, de la Torre C. Lung involvement in pyoderma gangrenosum: a case report and review of the literature. Article in Spanish. Actas Dermosifiliogr. 2011;102(5):373-377. doi: 10.1016/j.ad.2010.12.008 21397890

[8] Xu P, Cai Y, Ying X, Shi S, Song W. A case of persistent fever, cutaneous manifestations and pulmonary and splenic nodules: clinical experience and a literature review. Intern Med J. 2019;49(2):247-251. doi: 10.1111/imj.14201 30754076 PMC6849849

[9] Deregnaucourt D, Buche S, Coopman S, Basraoui D, Turck D, Delaporte E. Pyoderma gangrenosum with lung involvement treated with infliximab. Article in French. Ann Dermatol Venereol. 2013;140(5):363-366. doi: 10.1016/j.annder.2013.01.428 23663708

[10] Gade M, Studstrup F, Andersen AK, Hilberg O, Fogh C, Bendstrup E. Pulmonary manifestations of pyoderma gangrenosum: 2 cases and a review of the literature. Respir Med. 2015;109(4):443-450. doi: 10.1016/j.rmed.2014.12.016 25622759

[11] Vacas AS, Torre AC, Bollea-Garlatti ML, Warley F, Galimberti RL. Pyoderma gangrenosum: clinical characteristics, associated diseases, and responses to treatment in a retrospective cohort study of 31 patients. Int J Dermatol. 2017;56(4):386-391. doi: 10.1111/ijd.13591 28295267

[12] Brooklyn TN, Dunnill MG, Shetty A, Infliximab for the treatment of pyoderma gangrenosum: a randomised, double blind, placebo controlled trial. Gut. 2006;55(4):505-509. doi: 10.1136/gut.2005.074815 16188920 PMC1856164

